# The “develOpment of metabolic and functional markers of Dementia IN Older people” (ODINO) Study: Rationale, Design and Methods

**DOI:** 10.3390/jpm10020022

**Published:** 2020-04-09

**Authors:** Anna Picca, Daniela Ronconi, Hélio J. Coelho-Junior, Riccardo Calvani, Federico Marini, Alessandra Biancolillo, Jacopo Gervasoni, Aniello Primiano, Cristina Pais, Eleonora Meloni, Domenico Fusco, Maria Rita Lo Monaco, Roberto Bernabei, Maria Camilla Cipriani, Emanuele Marzetti, Rosa Liperoti

**Affiliations:** 1 Fondazione Policlinico Universitario “Agostino Gemelli” IRCCS, 00168 Rome, Italy; anna.picca@guest.policlinicogemelli.it (A.P.); riccardo.calvani@guest.policlinicogemelli.it (R.C.); jacopo.gervasoni@policlinicogemelli.it (J.G.); anielloprim@gmail.com (A.P.); cristina.pais@guest.policlinicogemelli.it (C.P.); meloni.eleonora@hotmail.it (E.M.); domenico.fusco@policlinicogemelli.it (D.F.); mariarita.lomonaco@policlinicogemelli.it (M.R.L.M.); mariacamilla.cipriani@policlinicogemelli.it (M.C.C.); rosa.liperoti@policlinicogemelli.it (R.L.); 2 Università Cattolica del Sacro Cuore, 00168 Rome, Italy; danielaronconi88@gmail.com (D.R.); coelhojunior@hotmail.com.br (H.J.C.-J.); 3 Department of Chemistry, Sapienza Università di Roma, 00185 Rome, Italy; federico.marini@uniroma1.it; 4 Department of Physical and Chemical Sciences, Università degli Studi dell’Aquila, 67100 L’Aquila, Italy; alessandra.biancolillo@univaq.it

**Keywords:** aging, biomarkers, cytokines, cognitive decline, Alzheimer’s disease, metabolomics, neuroinflammation, multivariate analysis, physical performance, person-tailored

## Abstract

Mild cognitive impairment (MCI), also termed mild neurocognitive disorder, includes a heterogeneous group of conditions characterized by declines in one or more cognitive domains greater than that expected during “normal” aging but not severe enough to impair functional abilities. MCI has been associated with an increased risk of developing dementia and even considered an early stage of it. Therefore, noninvasively accessible biomarkers of MCI are highly sought after for early identification of the condition. Systemic inflammation, metabolic perturbations, and declining physical performance have been described in people with MCI. However, whether biological and functional parameters differ across MCI neuropsychological subtypes is presently debated. Likewise, the predictive value of existing biomarkers toward MCI conversion into dementia is unclear. The “develOpment of metabolic and functional markers of Dementia IN Older people” (ODINO) study was conceived as a multi-dimensional investigation in which multi-marker discovery will be coupled with innovative statistical approaches to characterize patterns of systemic inflammation, metabolic perturbations, and physical performance in older adults with MCI. The ultimate aim of ODINO is to identify potential biomarkers specific for MCI subtypes and predictive of MCI conversion into Alzheimer’s disease or other forms of dementia over a three-year follow-up. Here, we describe the rationale, design, and methods of ODINO.

## 1. Introduction

Mild cognitive impairment (MCI), also termed mild neurocognitive disorder according to the Diagnostic and Statistical Manual of Mental Disorders, Fifth Edition (DSM-5) [1], is a condition characterized by a decline in cognitive function that is greater than that expected for the individual’s age and education level but not severe enough to compromise engagement in daily activities [2]. From a neuropsychological perspective, MCI involves reduced cognitive abilities (non-amnestic subtype) in one (single domain) or more (multiple domains) domains or a reduced ability to recall stored information (amnestic subtype) [3]. Other cognitive domains including language, visuospatial function, complex attention, and executive functions may also be affected [3]. Noticeably, people diagnosed with MCI, especially those with the amnestic subtype (aMCI), have a 10-fold increased risk of progression toward Alzheimer’s disease (AD) or other forms of dementia [4]. Therefore, establishing whether MCI is indeed a prodromal stage of dementia and understanding the mechanisms of its progression are necessary for the early take-in-charge of affected individuals and for the implementation of preventive interventions [5,6]. To this aim, biomarkers capable of identifying persons with MCI, especially those at higher risk of developing AD or other forms of dementia, are highly sought after.

Signs of neuroinflammation (e.g., detection of inflammatory cytokines in the proximity of ß-amyloid deposits and neurofibrillary tangles), whole-body metabolic perturbations, and declining physical performance (e.g., slow gait speed, impaired performance on dual-task tests) have been documented in people with MCI [7,8]. However, the existence of specific patterns of biological and functional markers across MCI subtypes and their predictive value for the conversion of MCI into dementia are debated.

Multi-marker approaches covering different physiological domains are increasingly implemented for the appraisal of complex and dynamic conditions [9,10,11]. These approaches are conceptualized within the notion of allostatic load, that is the exposure of cells and biological systems to recurrent or chronic stressors inflicting cumulative damage [12]. Within this paradigm, biomarkers represent endophenotypes of physiological dysregulation that may support diagnosis, tracking, clinical and therapeutic decision-making, and verification of the efficacy of an intervention before it is clinically detectable. In this scenario, the quantification of specific parameters coupled with ad hoc multivariate statistical approaches may allow identifying patterns of biomarkers that can be applied at the individual level as a measure of departure from his/her “normal operating conditions” [13]. 

The “develOpment of metabolic and functional markers of Dementia IN Older people” (ODINO) study has been conceived as an innovative multi-dimensional investigation in which clinical, neuropsychological, functional, and biological parameters will be analyzed through ad hoc statistical analyses to provide a comprehensive characterization of MCI subtypes. Biological and functional markers will be tested for their ability to predict MCI conversion into AD and other forms of dementia over a three-year follow-up.

## 2. Materials and Methods

### 2.1. Study Design and Population

The protocol of this observational study was approved by the Ethics Committee of the Fondazione Policlinico Universitario “Agostino Gemelli” IRCCS (Rome, Italy) (protocol #230/19). A convenience sample of 120 participants will be enrolled in ODINO. The study will be carried out through a two-step analytical process: (1) collection of clinical data, evaluation of cognitive and physical performance, and analysis of biological markers to evaluate patterns of physical performance, systemic inflammation, and metabolic perturbations in older adults with MCI, and (2) a longitudinal three-year follow-up to obtain indications of biomarkers associated with the conversion of MCI into dementia.

Participant recruitment will take place at the Fondazione Policlinico Universitario “Agostino Gemelli” IRCCS under the coordination of the outpatient clinic of the Department of Geriatrics. Participants will be recruited by convenience and asked about their willingness to participate in the study. Candidates aged ≥65 and ≤85 years with a diagnosis of MCI according to the criteria of the National Institute on Aging-Alzheimer’s Association [14] will be considered eligible for enrolment. After obtaining written informed consent, participants will be stratified in MCI subtypes (i.e., amnestic single domain, non-amnestic single domain, amnestic multi-domains, and non-amnestic multi-domains).

Exclusion criteria will be: active treatment for cancer or cancer diagnosis (except for non-melanoma skin cancer), severe knee or hip osteoarthritis limiting mobility, inflammatory diseases (e.g., rheumatoid arthritis, vasculitis, autoimmune disorders, inflammatory bowel disease), stroke with upper and/or lower extremity involvement, Parkinson’s disease or other neurological disorders likely to interfere with physical function, major psychiatric illnesses, sleep disorders, heart failure New York Heart Association (NYHA) class III–IV, respiratory insufficiency requiring supplemental oxygen, and use of long-acting benzodiazepines or antipsychotic drugs. Temporary exclusion criteria will be acute illnesses (e.g., infections, re-exacerbation of chronic obstructive pulmonary disease), major surgery, and traumata.

Participant assessment will be carried out at the geriatric outpatient clinic of the Fondazione Policlinico Universitario “Agostino Gemelli” IRCCS during four visits at baseline and every 12 months over a three-year follow-up. The activities expected at each visit will be completed over three days within one week (Table 1).

### 2.2. General Characteristics

Information on age, sex, smoking habit, alcohol consumption, comorbid conditions, and medications will be recorded by an attending physician through a structured interview and careful review of medical records. Vital signs will also be assessed. Standard blood biochemistry will be carried out by the centralized diagnostic laboratory of the Fondazione Policlinico Universitario “Agostino Gemelli” IRCCS.

### 2.3. Neuropsychological Assessment and Cognitive Evaluation

Participants will receive a comprehensive neuropsychological assessment and cognitive evaluation by means of the clinical dementia rating (CDR) scale [15] and a battery of neuropsychological tests exploring global cognitive function (mini-mental state examination, MMSE) [16], verbal learning and episodic memory (Rey auditory verbal learning test, RAVLT) [17], verbal short-term memory and verbal working memory (digit span) [18], visuospatial short-term memory and visuospatial working memory (Corsi span) [19,20], visuospatial abilities and praxis (copying drawings; copying drawings with landmarks; Rey-Osterrieth complex figure copy) [21], language (phonological verbal fluency task, semantic verbal fluency task, nouns naming test, and verbs naming test) [22], and attention and executive functions (Stroop color word interference test, multiple features target cancellation test, MFTC) [23,24]. Mood will be assessed through the 15-item geriatric depression scale (GDS-15) [25].

### 2.4. Anthropometry and Body Composition

Body mass and height will be measured by means of a medical graded weight scale with a stadiometer. Body mass index (BMI) will be calculated as the ratio between body mass (kg) and the square of height (m^2^).

A flexible and inextensible anthropometric tape will be used to measure waist circumference (WC), hip circumference (HC), and mid-arm circumference (MAC). The waist-to-hip ratio will be calculated. For these measurements, participants will be requested to wear light clothes and to stay in standing position, head held erect, eyes forward, with arms relaxed at the sides of the body, and feet kept together. WC will be taken at the mid-point between the last floating rib and the highest point of the iliac crest. HC will be measured at the highest point of the buttocks. MAC will be taken at the mid-point between the elbow and the deltoid muscle [26].

Bone mineral density and appendicular lean mass will be measured by dual X-ray absorptiometry (DXA) on a Hologic^®^ Discovery A (Hologic, Inc., Bedford, MA, USA), as previously described [27].

### 2.5. Assessment of Muscle Strength, Physical Function, and Disability Status

Upper and lower-limb muscle strength will be measured by isometric handgrip strength (IHG) and isokinetic analysis, respectively. IHG will be measured using a Jamar handheld hydraulic dynamometer (Patterson Medical Products, Inc., Cincinnati, OH, USA) [28]. For the test, participants will remain seated on a standard chair with shoulders abducted, and the elbow flexed at 90° and near to the trunk, and the wrist in a neutral position (thumb up). The contralateral arm will remain relaxed under the thigh [29]. IHG will be measured during four s. Participants will be familiarized and warmed up before performing three maximal efforts with a one-min rest period. Encouragement to perform the test as quickly and forcefully as possible will be provided during the entire experiment. The maximal concentric isokinetic strength of knee extensors of the dominant side will be measured on a REV9000 isokinetic dynamometer (Technogym, Gambettola, Italy), as previously described [30]. Briefly, participants will be asked to produce their maximum force while extending the knee from 90° to 0° of flexion at 60°/s with a hip angle of 90°–100°. Two practice repetitions will be completed prior to three test repetitions. The maximal peak torque achieved will be used for the analysis.

The timed-up-and-go (TUG) test involves getting up from a chair, walking three meters around a marker placed on the floor, coming back to the same position, and sitting back on the chair. Participants will begin the test while wearing their regular footwear, with their back against the chair, arms resting on the chair’s arms, and with the feet in contact with the ground. A researcher will instruct them to, on the word “go”, get up and walk at a normal pace through the demarcation of three meters on the ground, turn, return to the chair, and sit down again. Timing will start when participants get up from the chair and will stop when their back touches the backrest of the chair again [31]. After completing the standard TUG test, participants will be asked to perform TUG combined with a verbal fluency task (i.e., naming as many animals as they can remember), with a motor task (i.e., carrying a full cup of water), and with both cognitive and motor tasks (i.e., performing the verbal fluency test while carrying a full cup of water) [32].

The short physical performance battery (SPPB) is composed of three subtasks: standing balance, usual gait speed, and the five-repetition chair-stand test [33]. For the standing balance test, participants will be asked to stand in three progressively more difficult positions for 10 s each: a side-by-side feet standing position, a semi-tandem position and a full-tandem position. Gait speed will be measured over a four m course at the person’s usual pace. The faster of two trials will be used for the analysis. For the five-repetition chair-stand test, participants will be asked to perform five repetitions of standing up and sitting down from a chair without using hands and the performance will be timed. Each of the three SPPB subtasks will be categorized into a five-level score, with zero representing the inability to do the test and four corresponding to the highest level of performance.

The six-min walk test will be performed according to the guidelines of the American Thoracic Society [34]. The test will be conducted indoors on a 30-m track. After remaining seated for 15 min, participants will be requested to walk on the track as fast as possible for six min. The test will be interrupted if participants show any sign of chest pain, intolerable dyspnea, leg cramps, staggering, diaphoresis, a pale or ashen appearance, or any other complaint. The distance walked (m) will be used for the analysis.

Disability status will be assessed through the activities of daily living (ADL) and instrumental ADL (IADL) scales [35,36].

### 2.6. Measurement of Physical Activity Levels and Energy Expenditure

Free-living physical activity levels and energy expenditure will be quantified through a SenseWear^®^ armband (SWA, BodyMedia, Inc., Pittsburgh, PA, USA) over seven consecutive days. The SWA is a body monitor wearable on the back of the arm that enables the continuous monitoring of physical activity at low intensities and during unstructured or intermittent activities. The SWA utilizes a unique combination of sensors to measure the amount of heat being dissipated by the body as well as the skin and near-armband temperature. Measures of galvanic skin response to physical and emotional stimuli are also recorded. A two-axis accelerometer tracks the arm movements and provides information about the body position. Wireless transmission, communication, and wired data download are ensured by a radio and a data port. All information collected is integrated and processed by software using proprietary algorithms according to the participant’s characteristics (sex, age, height, and weight) to provide minute-by-minute estimates of energy expenditure during different levels of physical activity [37].

### 2.7. Collection of Blood Samples

Blood will be drawn in the morning by venipuncture of the median cubital vein. For plasma separation, blood will be collected using tubes containing ethylenediaminetetraacetic acid (EDTA) and will subsequently be centrifuged at 1000× *g* for 15 min at 4 °C. For serum separation, blood samples will be collected in tubes without EDTA and will be kept at room temperature for 30 min to allow clotting. Afterward, samples will be centrifuged as described above. Aliquots will be prepared from the upper clear fraction (plasma or serum) and stored at −80 °C until analysis.

### 2.8. Determination of Biomarkers of Inflammation and Neurodegeneration

A biomarker panel has been designed based on previous investigations by our group in older populations, including older adults with neurodegenerative conditions [10,38,39,40,41,42,43]. Markers of systemic inflammation will be assayed as described elsewhere [40]. Briefly, a set of 27 pro- or anti-inflammatory mediators (e.g., cytokines, chemokines, and growth factors) will be measured in serum samples using the Bio-Plex Pro™ Human Cytokine 27-plex Assay kit (#M500KCAF0Y, Bio-Rad, Hercules, CA, USA) (Table 2). Experiments will be run on a Bio-Plex^®^ System with Luminex xMap Technology (Bio-Rad) and data will be acquired on the Bio-Plex Manager Software 6.1 (Bio-Rad) with instrument default settings. Outliers will be automatically removed by optimization of standard curves across all analytes and results will be obtained as concentration (pg/mL).

Traditional and automated enzyme-linked immunosorbent assays will be run to quantify serum levels of amyloid beta (aa1-42) (#DAB142, R&D Systems, Minneapolis, MN, USA), neurofilament light polypeptide (#SPCKB-PS-002448-000190, R&D Systems), Tau (#NBP2-62749, Novus Biologicals, Littleton, CO, USA), and Tau [p Ser739] (#NBP2-66711, Novus Biologicals) proteins according to the manufacturer’s instructions.

### 2.9. Measurement of Plasma Fatty Acid Concentrations

A panel of 14 fatty acids that were previously associated with AD [44] will be measured in plasma by a gas chromatography-electron ionization-mass spectrometry (GC-EI-MS) validated methodology (Eureka One Lab Division Kit, code GC75010; Ancona, Italy) according to the manufacturer’s instructions (Table 3).

Following extraction and washing, samples will be treated with a derivatizing solution for 15 min at 100 °C, diluted and directly injected into the GC-MS. GC-MS analyses will be performed on a Trace GC Ultra (Thermo Fisher Scientific, Waltham, MA, USA) equipped with Durabond HP-88 column, 100 m × 0.25 mm × 0.2 μm film thickness (Agilent Technologies, Santa Clara, CA, USA), and connected to an ISQ mass spectrometer (Thermo Fisher Scientific). One μL of the sample will be injected in split mode (1:10 ratio), with injector temperature set at 250 °C. Helium will be used as carrier gas and the flow-rate will be maintained constant at 1 mL/min. The initial oven temperature of 100 °C will be held for 1 min, then raised to 220 °C at 10 °C/min, and maintained for 4 min. Afterward, the oven temperature will be increased up to 240 °C at 10 °C/min and held for 10 min. The mass transfer line will be maintained at 270 °C and the ion source at 200 °C. Analyses will be performed in the selected ion monitoring (SIM) mode. Ions monitored in the analysis are shown in Table 3. The analytical process will be monitored using fatty acid controls (code GC75019, level 1 and level 2) manufactured by Eureka One Lab Division.

### 2.10. Measurement of Serum Concentrations of Amino Acids and Derivatives

The serum concentration of a panel of 37 amino acids and derivatives will be determined by ultraperformance liquid chromatography/MS (UPLC/MS), as described elsewhere [9]. The panel has been chosen based on previous work by our group in older populations [9,41,45,46] and will include: 1-methylhistidine, 3-methylhistidine, 4-hydroxyproline, α-aminobutyric acid, β-alanine, β-aminobutyric acid, γ-aminobutyric acid, alanine, aminoadipic acid, anserine, arginine, asparagine, aspartic acid, carnosine, citrulline, cystathionine, cystine, ethanolamine, glutamic acid, glycine, histidine, isoleucine, leucine, lysine, methionine, ornithine, phenylalanine, phosphoethanolamine, phosphoserine, proline, sarcosine, serine, taurine, threonine, tryptophan, tyrosine, and valine.

Briefly, 50 μL of the sample will be added to 100 μL 10% (w/v) sulfosalicylic acid containing an internal standard mix (50 μM) (Cambridge Isotope Laboratories, Inc., Tewksbury, MA, USA). The mixture will be centrifuged at 1000× *g* for 15 min. Seventy μL of borate buffer and 20 μL of AccQ Tag reagents (Waters Corporation, Milford, MA, USA) will be added to 10 μL of the obtained supernatant and heated at 55 °C for 10 min. Next, samples will be loaded onto a CORTECS UPLC C18 column 1.6 μm, 2.1 mm × 150 mm (Waters Corporation) for chromatographic separation (ACQUITY H-Class, Waters Corporation). Elution will be accomplished at 500 μL/min flow-rate with a linear gradient (9 min) from 99:1 to 1:99 water 0.1% formic acid/acetonitrile 0.1% formic acid. Finally, analytes will be detected on an ACQUITY QDa single quadrupole mass spectrometer equipped with an electrospray source operating in positive mode (Waters Corporation). Amino acid controls manufactured by the MCA laboratory of the Queen Beatrix Hospital (Winterswijk, The Netherlands) will be used to monitor the analytic process.

### 2.11. Statistical Analysis

Since no studies have explored the multi-marker profile of MCI chosen in ODINO, no power analysis could be run for sample size calculation. Based on the number of older persons diagnosed with MCI at our center, we estimate that 120 participants will be enrolled over one year. The rate of losses to follow-up over three years is expected to be 20%, leaving a total of 100 cases from whom all variables of interest will be collected. Participants will be censored at the end of follow-up, at the date of conversion to dementia, or at the date of death, as applicable.

Descriptive statistics will be run for all variables. After ascertainment of data distribution, comparisons of variables of interest among MCI subtypes will be performed via analysis of variance (ANOVA) or Kruskal –Wallis H for continuous variables, as appropriate. Categorical variables will be compared through χ^2^ statistics. Survival analyses (Kaplan–Meier and Cox regression) will be used to investigate the impact of clinical variables on conversion to dementia. Descriptive statistics will be performed using SAS version 9.2 (SAS Institute Inc., Cary, NC, USA). 

In the cross-sectional stage of the study, to characterize MCI subtypes, different classification strategies will be enacted. First, a purely discriminant approach will be adopted. In discriminant classification, data are used to build a predictive model aiming at assigning each individual to one specific group (in this case, any of the MCI subtypes). To this aim, a classification strategy based on partial least squares discriminant analysis (PLS-DA) will be adopted [47]. PLS-DA offers the advantage of processing datasets containing a high number of variables even if they are highly correlated with one another. The analysis of the whole dataset, which encompasses multi-block data, will be performed through the recently developed sequential and orthogonalized covariance selection (SO-CovSel) [48]. The method, which allows a highly parsimonious variable selection, was used by our group for the identification of a gut microbial, inflammatory and metabolic fingerprint in older adults with physical frailty & sarcopenia [41].

To rule out the possibility of chance correlations and to estimate the reliability of predictive models, a thorough validation process by means of double cross-validation (DCV) and permutation tests will be operated. DCV consists of two nested loops of cross-validation; the external loop is used to unbiasedly estimate the predictive ability of the model parameters that are optimized on the basis of the internal loop. The classification ability of the optimal model is then expressed by means of various figures of merit, such as the number of misclassifications (NMC), the area under the receiver operating characteristic (AUROC) curve, and the discriminant Q2 (DQ2), the distributions of which under the null hypothesis are estimated via permutation tests [49]. This allows the establishment of the statistical significance of the observed discrimination. A detailed description of PLS-DA, SO-CovSel, and DCV procedures may be found elsewhere [9,41,43].

Classification will be repeated with a modeling approach based on soft independent modeling of class analogies (SIMCA) [50], in order to assess the degrees of similarity among MCI subtypes. Modeling approaches use data to define which experimental profiles are to be expected from individuals belonging to a particular category (i.e., the so-called model space). Accordingly, a class model allows the prediction of how likely it is for an individual to belong to a class or not, on the basis of the measurements considered. Since each category (MCI subtype) will be modeled independently from the others, the results of this analytical stage will provide information on the effectiveness of the characterization of different MCI subtypes. The analysis will also indicate how likely it is for specific subtypes of participants to be confounded with one another. A SIMCA model was recently built by our group to verify the accuracy of the classification of older adults with Parkinson’s disease based on extracellular vesicle cargo [43].

In the longitudinal phase of the study, multilevel ANOVA (MANOVA) coupled with simultaneous component analysis (SCA) or with PLS-DA will be used to characterize the time dynamics of participants over the follow-up [51]. MANOVA may be considered to be equivalent to multivariate ANOVA for repeated measurements. In such a configuration, SCA, which under the constraints of MANOVA is identical to principal component analysis, or PLS-DA allows applying the method to data matrices containing a high number of possibly correlated variables. The statistical significance of MANOVA will be assessed non-parametrically by permutation tests. Multivariate statistics will be conducted using functions written in-house and run under the Matlab environment (The Mathworks, Natick, MA, USA).

## 3. Discussion

The possibility that MCI, at least in some variants, may be a prodromal stage of dementia has ignited a great deal of research primarily aimed at testing possible strategies to impede MCI progression [52]. However, the clinical heterogeneity of MCI and its multifactorial pathogenesis have been major hurdles in identifying meaningful biomarkers and devising effective interventions.

Different cognitive domains may be affected in MCI, which has allowed the clinical identification of various subtypes. In the aMCI, memory loss is predominant and shows a higher risk for further conversion to AD [53]. Non-amnestic MCI subtypes are characterized by impairments in cognitive domains other than memory and have a greater propensity to convert into other forms of dementia, such as diffuse Lewy body dementia and vascular dementia [53]. Both aMCI and naMCI variants can be further categorized into single- and multi-domain subtypes, depending on the number of cognitive domains affected. The distinction between aMCI and naMCI is not only based on neuropsychological parameters, but is sustained by specific brain structural characteristics, primarily involving the hippocampus, the cortical thickness, and the entorhinal cortex [54,55,56,57].

The development of biomarkers able to capture complex phenomena, such as MCI and dementia, are highly sought after. Several investigations have been conducted to evaluate candidate biomarkers for MCI. However, these studies investigated differences between MCI and healthy controls, whereas none of them explored distinct features across MCI subtypes [58,59].

The development of cost-effective omics platforms enabling the simultaneous analysis of a vast repertoire of biological mediators in biofluids has shown great value in providing a comprehensive read-out of the environmental and clinical disturbances affecting cell homeostasis in different settings [11]. Metabolomics analyses of the cerebrospinal fluid (CSF) allowed differentiating MCI from dementia in older people, a task in which traditional biomarkers of AD such as Aβ42 failed [60]. Core CSF biomarkers including total (T-Tau) and phosphorylated Tau (P-Tau) protein, Aβ42, and neurofilament light polypeptide have been strongly associated with AD, such that their clinical implementation for diagnostic purposes has been suggested [61]. However, biomarkers specific for MCI are still missing.

Recent advances in lipidomics suggest that fatty acid dysmetabolism and imbalance in fatty acid lipidome are involved in the initiation and progression of several neurodegenerative diseases, including AD [62]. Indeed, fatty acid β-oxidation and its byproducts impact immune cell function, thereby possibly contributing to neuroinflammation [62]. However, no studies have yet addressed whether specific lipid markers distinguish aMCI and naMCI and predict their conversion into dementia. Furthermore, an investigation involving eight prospective cohorts with over 20,000 participants found an inverse association between serum concentrations of branch-chained amino acids and incident AD [63]. Similar to lipids, it is presently unclear whether specific patterns of circulating amid acids are selectively associated with MCI subtypes.

Inflammatory cytokines, including interleukin (IL) 1β, IL6 and tumor necrosis factor-alpha (TNF-α), are involved in local inflammation triggered by amyloid plaque deposition and may induce neurotoxicity when produced chronically, favoring the generation of Aβ peptides [64]. Therefore, a role for these cytokines in inter- and intracellular signaling in microglia and astrocytes has been hypothesized in AD [65]. Interestingly, peripheral inflammatory markers associated with MCI are distinct from those found in patients with AD [66]. Though, no conclusive data are presently available.

Since markers pertaining to a single domain (e.g., inflammatory rather than metabolic) may fail at capturing the intertwined relationship between local and systemic changes, we decided to combine metabolomics analysis and immunoassays to characterize the metabolic and inflammatory profile of older adults with MCI. A biomarker panel has been designed based on previous investigations by our group in older populations, including older adults with neurodegenerative conditions [10,38,39,40,41,42,43]. This approach was also guided by the recent advances made in the field of geroscience [67]. To gain insights into the etiology of the MCI subtypes, the panel also includes the analysis of circulating levels of T-Tau and P-Tau protein, Aβ42, and neurofilament light polypeptide [61].

Other lines of evidence indicate that specific impairments in physical performance are associated with MCI. In particular, people with aMCI show significant decreases in gait speed and increases in stride time and time variability when changing from a single- to a dual-task [68,69]. Notably, poor gait performance, particularly under dual-tasking, has been proposed as a motor signature of aMCI [8]. It is noteworthy that the co-occurrence of cognitive complaints and slow gait, a condition referred to as motoric cognitive risk syndrome, identifies individuals at especially high risk to progress to dementia [70,71]. Along similar lines, low muscle strength of upper and lower extremities has shown to increase the risk of MCI progression into AD [72]. The neurophysiologic substratum of reduced physical performance in MCI has its roots in the existence of neuronal networks involved in both cognition and lower extremity function. Indeed, atrophy and amyloid deposition in a network encompassing the dorsolateral prefrontal cortex, cingulate gyrus, parietal association areas, basal ganglia, and medial temporal lobes (particularly the hippocampus) are thought to mediate the relationship between cognitive function and physical performance [73]. Thus, individuals who exhibit both cognitive and motor deficits may have greater underlying brain damage [71]. This implies that the simultaneous analysis of cognitive and physical function may help identify a subset of MCI persons at greater risk of conversion to dementia [71,74]. The observation that low muscle mass is commonly observed in conjunction with cognitive impairment [75,76] suggests that the inclusion of body composition analysis might further refine the identification of people more likely to progress from MCI to dementia. Indeed, muscle loss and cognitive dysfunction share a number of predisposing factors, including inflammation, oxidative stress, insulin resistance, and an inactive lifestyle [76]. With regard to the latter, studies have shown that engagement in regular physical activity is negatively associated with the risk of developing dementia [77,78,79]. Furthermore, findings from a systematic review and meta-analyses indicated that an increase of 500 kcal or 10 MET-h per week was associated with a 10% decrease in the risk of dementia [79].

## 4. Conclusions

The ODINO study was conceived as an innovative multi-dimensional investigation aimed at exploring biological and physical performance signatures of MCI subtypes. Measures of inflammatory/metabolic markers and physical performance will be integrated through advanced multivariate statistical analyses to gain insights into the heterogeneity of MCI subtypes and their risk to progress toward dementia. The results obtained from the ODINO study and their comparison with those collected from a thoroughly characterized cohort of non-MCI older adults with similar age and sociodemographic characteristics [9,10,11] may enable discerning pathways involved in “physiologic” age-related cognitive decline from those implicated in the progression of MCI to early dementia. This knowledge will, therefore, pave the way for the clinical implementation of composite biomarkers of MCI. Our results will also allow the possible identification of therapeutic targets amenable for person-tailored interventions that may hold people with MCI back from the doorstep of dementia.

## Figures and Tables

**Table 1:** Visit schedule and related activities.

Activity	Visit 1 (Baseline)	V2 (12 Months)	V3 (24 Months)	V4 (36 Months)
Informed Consent	x			
Sociodemographic Characteristics	x	x	x	x
Medical History	x	x	x	x
Medication Inventory	x	x	x	x
CDR Scale	x	x	x	x
MMSE	x	x	x	x
RAVLT	x	x	x	x
Digit Span	x	x	x	x
Corsi Span	x	x	x	x
Visuospatial Abilities and Praxis	x	x	x	x
Language	x	x	x	x
Attention and Executive Functions	x	x	x	x
GDS-15	x	x	x	x
Anthropometry	x	x	x	x
Body Composition	x	x	x	x
Muscle Strength	x	x	x	x
TUG	x	x	x	x
SPPB	x	x	x	x
6MWT	x	x	x	x
ADL	x	x	x	x
IADL	x	x	x	x
7-Day Activity and EE Monitor	x	x	x	x
Blood Draw	x	x	x	x

The activities of each visit will be completed over three days within one week. Abbreviations: CDR, clinical dementia rating; MMSE, mini-mental state examination; RAVLT, Rey auditory verbal learning test; GDS-15, geriatric depression scale 15 items; TUG, timed-up-and-go; SPPB, short physical performance battery; 6MWT, six-min walking test; ADL, activities of daily living; IADL, instrumental activities of daily living; EE, energy expenditure.

**Table 2:** Serum inflammatory biomolecules assayed by multiplex immunoassay.

Biomarker Class	Components of the Biomarker Panel
Cytokines	IFNγ, IL1β, IL1Ra, IL2, IL4, IL5, IL6, IL7, IL8, IL9, IL10, IL12, IL13, IL15, IL17, TNF-α
Chemokines	CCL5, CCL11, IP-10, MCP-1, MIP-1α, MIP-1β
Growth Factors	FGF-β, G-CSF, GM-CSF, PDGF-BB

Abbreviations: IFN, interferon; IL, interleukin; IL1Ra, interleukin 1 receptor agonist; TNF-α, tumor necrosis factor-alpha; CCL, C-C motif chemokine ligand; IP: interferon-induced protein; MCP-1: monocyte chemoattractant protein 1; MIP: macrophage inflammatory protein; FGF, fibroblast growth factor; G-CSF, granulocyte colony-stimulating factor; GM-CSF, granulocyte macrophage colony-stimulating factor; PDGF-BB, platelet-derived growth factor BB.

**Table 3:** Plasma fatty acids assayed by gas chromatography-electron ionization-mass spectrometry.

Fatty Acid	Fragments
Tetradecanoic acid	74.1; 87.0; 143.0
Hexadecanoic acid	227.0; 270.0
Cis-9-hexadecenoic acid	55.0; 81.0; 96.0; 237.0
Heptadecanoic acid (internal standard)	74.1; 87.0; 284.0
Octadecanoic acid	75.0; 255.0; 298.0
Cis-9-octadecenoic acid	81.1; 96.0; 264.0
Trans-9-octadecenoic acid	81.1; 96.0; 264.0
All cis 9,12-octadecadienoic acid	67.0; 81.1; 95.0
Trans-9,trans-12-octadecadienoic acid	67.0; 81.1; 95.0
All cis 9,12,15 octadecatrienoic acid	67.0; 79.1; 93.0; 95.0
All cis 6,9,12 octadecatrienoic acid	67.0; 79.1; 93.0; 95.0
All cis 8,11,14 eicosatrienoic acid	67.0; 79.1; 93.0
All cis 5,8,11,14 eicosatetraenic acid	67.0; 79.1; 91.0
All cis 5,8,11,14,17 eicosapentaenoic acid	67.0; 79.1; 91.0; 105.0
